# Effects of Pyrazine Derivatives and Substituted Positions on the Photoelectric Properties and Electromemory Performance of D–A–D Series Compounds

**DOI:** 10.3390/ma11102063

**Published:** 2018-10-22

**Authors:** Xuejing Song, Lingqian Kong, Hongmei Du, Xiangyu Li, Hanlin Feng, Jinsheng Zhao, Yu Xie

**Affiliations:** 1 College of Chemical Engineering and Energy Technology, Dongguan University of Technology, Dongguan 523100, China; sxj_jing113@163.com; 2 Department of Chemistry and Chemical Engineering, Liaocheng University, Liaocheng 252059, China; duhongmei@lcu.edu.cn (H.D.); LXY201620@126.com (X.L.); fhl2660170344@163.com (H.F.); 3 Dongchang College, Liaocheng University, Liaocheng 252059, China; lingqiankong@126.com; 4 Key Laboratory of Jiangxi Province for Persistant Pollutants Control and Resources Recycle, Nanchang Hangkong University, Nanchang 330063, China

**Keywords:** pyrazine, quinoxaline, benzocarbazole, donor-acceptor-donor, photoelectric properties, electromemory

## Abstract

Pyrazine derivatives quinoxaline and pyridopyrazine were selected as the acceptors, and benzocarbazole was used as the donor to synthesize four different D–A–D compounds. The results showed that 2,3-bis(decyloxy)pyridine[3,4-*b*]pyrazine (DPP) exhibited stronger electron-withdrawing ability than that of 2,3-bis(decyloxy)quinoxaline (DPx), because DPP possesses one more nitrogen (N) atom, resulting in a red-shift of the intramolecular charge transfer (ICT) absorption bands and fluorescent emission spectra for compounds with DPP as the acceptor compared with those that use DPx as the acceptor. The band-gap energy (Eg) of the four D–A–D compounds were 2.82 eV, 2.70 eV, 2.48 eV, and 2.62 eV, respectively, for BPC-2DPx, BPC-3DPx, BPC-2DPP, and BPC-3DPP. The solvatochromic effect was insignificant when the four compounds were in the ground state, which became significant in an excited state. With increasing solvent polarity, a 30–43 nm red shift was observed in the emissive spectra of the compounds. The thermal decomposition temperatures of the four compounds between 436 and 453 °C had very high thermal stability. Resistor-type memory devices based on BPC-2DPx and BPC-2DPP were fabricated in a simple sandwich configuration, Al/BPC-2DPx/ITO or Al/BPC-2DPP/ITO. The two devices showed a binary non-volatile flash memory, with lower threshold voltages and better repeatability.

## 1. Introduction

Organic optoelectronic materials are mainly divided into two major categories according to the size of their molecules: small organic molecule materials and polymer materials. Small organic molecule materials have received considerable attention because of their advantages in determined molecular mass, easy purification, high fluorescent quantum yield, high electroluminescence, and low driving voltage [1,2,3,4]. Researchers have developed a wide variety of organic optoelectronic materials whose performance can be adjusted through molecular design [5,6,7,8,9,10,11,12,13,14]. For example, their solubility can be adjusted by changing the length of the alkyl substituents [4,5,6], and their absorption spectrum, carrier mobility, and forbidden band width can be adjusted by changing the type of the substituent [7,8,9,10] and the link mode [11,12,13,14]. The combination of an electron-rich unit, i.e., an electron donor (D), with electron-donating ability, and an electron-deficient unit, i.e., an electron acceptor (A), with electron-withdrawing ability to construct a D–A–D structure is referred as the D–A strategy [11,12,13,14,15,16,17]. In recent years, the construction of new optoelectronic materials using the D–A method by selecting different donors and acceptors has become an important research topic. The combination of different donors and acceptors resulted in a large number of molecules with different structures, which expands the range of spectral absorption to satisfy the needs of different applications. Intramolecular charge transfer (ICT) occurs in a D–A molecule, and it affects the molecule’s highest occupied molecular orbital (HOMO), lowest unoccupied molecular orbital (LUMO), and band gap, thereby affecting various optoelectronic properties [11,12,13,14,15,16,17,18,19]. Combining D or A units with similar structures, changing the heteroatoms, or changing the substituents can affect the properties of D–A-type materials [7,8,9,10,20,21].

Commonly used donor units are benzothiophene [21], fluorene [22], carbazole [23], triphenylamine, and cyclopentadithiophene [24], and so on. Commonly used acceptor units are mainly heterocyclic rings containing O, N, or S, such as pyrrole [25], pyridine [26], benzopyrazine [9,10,11,12,13], thienopyrazine [27], pyridopyrazine [28], benzothiadiazole [29], benzotriazole [30], and phenanthroline [31] et al. Pyrazine is a diazine with two N atoms symmetrically positioned at the one and four positions. Pyrazine has a higher electron affinity than pyridine, which contains only one N atom. Thus, it is more suitable as an acceptor [25]. In addition, pyrazine is very easy to modify, thereby regulating its electron-withdrawing ability. For example, different substituents, such as alkyl chains, benzene rings [25], and thiophene rings [10,25], can be introduced at the two and three positions of pyrazine, and the pyrazine ring can be fused with an aromatic ring to obtain quinoxaline or pyridopyrazine [7,8,9,10,11,12,13,32,33]. Pyridine, quinoxaline, and pyridopyrazine contain one N atom, two N atoms, and three N atoms, respectively, and their electron-withdrawing ability gradually increase in this order (e.g., reduction potential versus Hg pool: −0.86 V (pyridopyrazine) > −1.10 V (quinoxaline) > −2.20 V (pyridine)) [33].

Researchers [22,25,32] have compared the optical and electrochemical properties of D–A photopolymers using pyridopyrazine, pyridine, and quinoxaline as the acceptors. The results showed that the ultraviolet-visible (UV-vis) ICT absorption band of the polymers using pyridopyrazine as the acceptor was shifted 30 nm in the longer wavelength direction compared with the polymer using pyridine or quinoxaline as the acceptor. The reason is that pyridopyrazine has a stronger electron-withdrawing ability than pyridine or quinoxaline. Gratzel et al. [7] constructed a variety of D–A structural compounds with pyrazine derivatives as the acceptor and compared the effect of electron-withdrawing ability of the acceptors, the substituents of acceptors, and the link mode between the donor and the acceptors on the optoelectronic properties of the obtained material. Park et al. [14] designed and synthesized two organic sensitizers with horizontally and vertically conjugated D–A–D structures using quinoxaline as the acceptor and triphenylamine as the donor. Dye-sensitized solar cells with high optoelectronic conversion efficiency (3.3% and 5.56% for horizontally and vertically conjugated structures, respectively) were obtained. Kai Pei et al. [8] studied D–A–π–A compounds with quinoxaline as the auxiliary acceptor, and indoline and triphenylamine as donors. The introduction of the auxiliary acceptor quinoxaline optimizes the energy level of the compound, expands the absorption range, promotes the distribution of electrons on the donor, and improves the photostability of the compound. Xuefeng Lu et al. [13] synthesized a series of D–A–D type compounds using pyrazine fused with different numbers of aromatic or thiophene rings as the acceptors, and using triphenylamine as the donor to achieve a controlled charge transfer.

**Figure 1:**
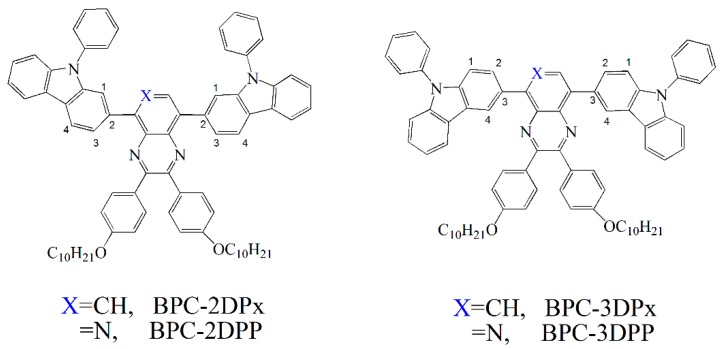
Structure of the four investigated D–A–D-type compounds.

In the design and synthesis of D–A-type optoelectronic materials, the effects of the donor and acceptor types, link sites, and substituents on the performance of the obtained optoelectronic materials have been intensively studied. However, the effect of the substitution position of the acceptor on the donor on the performance of photoelectric active compounds has rarely been reported. There are no reports of a D–A–D-type optoelectronic compounds with benzocarbazole as the donor and quinoxaline or pyridopyrazine as the acceptor. In this study, four compounds with the D–A–D-type structure were designed and synthesized using benzocarbazole as the donor and quinoxaline or pyridopyrazine as the acceptor (see Figure 1): 4,7-bis(9-phenyl-9*H*-carbazol-2-yl)-2,3-bis(decyloxy)quinoxaline (BPC-2DPx, the acceptor quinoxaline substituted at the 2-position of the donor benzocarbazole); 4.7-bis(9-phenyl-9*H*-carbazol-3-yl)-2,3-bis(decyloxy)quinoxaline (BPC-3DPx, the acceptor quinoxaline substituted at the 3-position of the donor benzocarbazole); 4,7-bis(9-phenyl-9*H*-carbazol-2-yl)-2,3-bis(decyloxy)pyridine [3,4-*b*]pyrazine (BPC-2DPP, the acceptor pyridopyrazine substituted at the 2-position of the donor benzocarbazole); and 4,7-bis(9-phenyl-9*H*-carbazol-3-yl)-2,3-bis(decyloxy)pyridine [3,4-*b*]pyrazine (BPC-3DPP, the acceptor pyridopyrazine substituted at the 2-position of the donor benzocarbazole). The relationship between the structure and properties of the four D–A–D compounds were studied by density functional theory (DFT), UV-vis spectroscopy, fluorescent spectroscopy, and cyclic voltammetry. The effects of the slight differences in the structures of the acceptors and the link sites between the acceptor and the donor on the optoelectronic properties of the resultant compounds were compared, as were the compounds’ structure–property relationships. In addition, BPC-2DPx and BPC-2DPP were chosen as electromemory materials, fabricating sandwich electromemory devices (Al/BPC-2DPx/ITO or Al/BPC-2DPP/ITO); their morphology, memory performance, and electromemory stability were investigated.

## 2. Results and Discussion

### 2.1. Synthesis of the Four Fluorescent Compounds

**Scheme 1:**
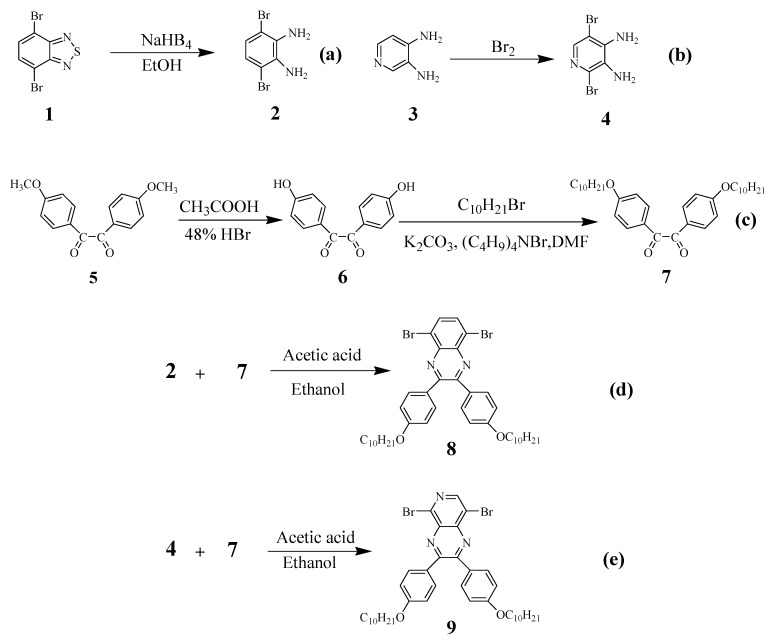
Synthesis routes of the acceptors quinoxaline and pyridopyrazine.

Studies have shown that the presence of large volumes of alkyl or alkoxy substituents on the acceptor is conducive to improve the optoelectronic conversion efficiency of the material [8]. Therefore, the acceptor DPx or DPP selected in this study contained two decyloxyphenyl substituents, e.g., 2,3-bis(4-decyloxybenzene)-5,8-dibromoquinoxaline (compound **8**) and 2,3-bis(4-decyloxybenzene)-5,8-dibromopyrido[3,4-*b*]pyrazine (compound **9**). The synthesis route is shown in Scheme 1d,e. The synthesis of acceptor compound **8** is divided into two steps. The first step was reduction of 4,7-dibromo-2,1,3-benzothiadiazole (compound **1**) by sodium borohydride (NaBH_4_) to obtain 3,6-dibromo-1,2-phenylenediamine (compound **2**). Secondly, compound **2** reacted with 4,4-dichenyloxybenzene (compound **7**) to obtain compound **8** [34]. The synthesis of acceptor compound **9** was also carried out in two steps. The first step was the bromination reaction of 3,4-diaminopyridine (compound **3**) to obtain 3,4-diamino-2,5-dibromopyridine (compound **4**). In the second step, compound **4** reacted with compound **7** to obtain compound **9** [35].

**Scheme 2:**
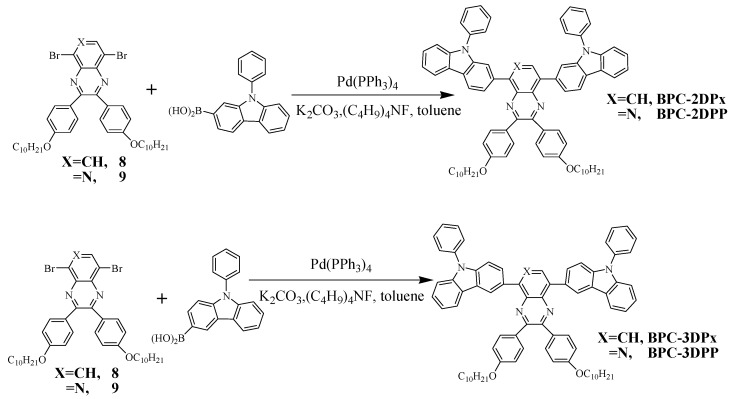
Synthesis routes of the four compounds.

The four D–A–D compounds were all obtained through one-step Suzuki coupling (Scheme 2) [6,9,13], and the yields were between 62–70%. The compounds BPC-2DPx and BPC-3DPx were obtained by Suzuki coupling reaction using compound **8** with 9-phenyl-9*H*-carbazol-2-yl-boronic acid (2-BPC) or 9-phenyl-9*H*-carbazol-3-yl-boronic acid (3-BPC) (Scheme 2). The compounds BPC-2DPP and BPC-3DPP were prepared in the same manner by replacing compound **8** with compound **9**.

### 2.2. Optimization of Molecular Energy Levels

**Figure 2:**
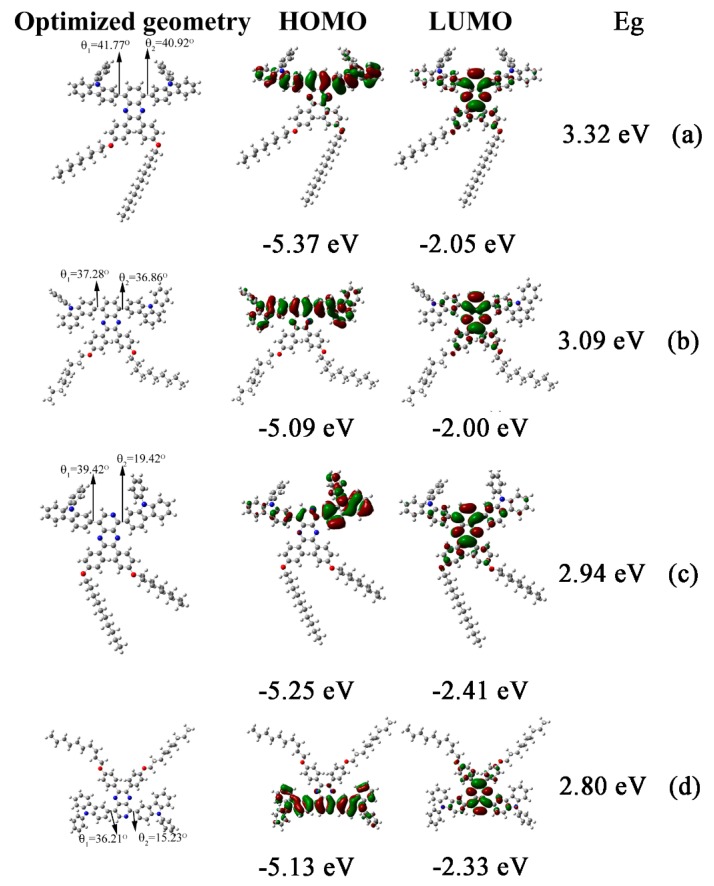
The frontier molecular orbital of 4,7-bis(9-phenyl-9*H*-carbazol-2-yl)-2,3-bis(decyloxy)quinoxaline (BPC-2DPx) (**a**), 4.7-bis(9-phenyl-9*H*-carbazol-3-yl)-2,3-bis(decyloxy)quinoxaline (BPC-3DPx) (**b**), 4,7-bis(9-phenyl-9*H*-carbazol-2-yl)-2,3-bis(decyloxy)pyridine[3,4-*b*]pyrazine (BPC-2DPP) (**c**) and 4,7-bis(9-phenyl-9*H*-carbazol-3-yl)-2,3-bis(decyloxy)pyridine[3,4-*b*]pyrazine (BPC-3DPP) (**d**).

The density functional theory (DFT) was used to optimize the structure of the compounds. The molecular structure after optimization and the HOMOs and LUMOs are shown in Figure 2.

As shown in Figure 2, the distributions of π electrons in the HOMO of the four compounds were mainly distributed on the benzocarbazole donor unit, while the excited electrons in the LUMO of the four compounds were mainly distributed on the acceptor moieties, including pyridopyrazine and quinoxaline. This indicates that ICT occurred from the ground state to the excited state [8,9,10,11,12,13], and the electrons were transferred from benzocarbazole to pyridopyrazine or quinoxaline. The alkoxy side chain had no obvious electron supply effect. DFT-based calculations were performed using Gaussian software at the level of theory B3LYP/6-31 g+(d) [13], and the calculated values of the HOMO level, LUMO level, and band-gap energy (*E*_g_) are shown in Figure 2. The HOMO levels in BPC-2DPx and BPC-2DPP only slightly differed, as did the HOMO levels in BPC-3DPx and BPC-3DPP. However, the LUMO levels of compounds with DPP as the acceptor were lower than those with DPx as the acceptor. This difference indicates that DPP had a stronger electron-withdrawing ability than DPx, which resulted in stronger ICTs, the red-shifted absorption spectra, and lower energy gaps.

Figure 2 also shows the dihedral angle data between the acceptor and the adjacent donor in the four compounds, enabling comparisons of their coplanarity. The dihedral angles of the two-substituted compounds were greater than those of the three-substituted isomers, indicating that the three-substituted compounds exhibited better coplanarity than the two-substituted compounds. In the case of DPx as the acceptor, there was no significant difference in the dihedral angles between the acceptor and the two donors, indicating that both BPC-2DPx and BPC-3DPx have good symmetry. However, in the case of DPP as the acceptor, the dihedral angles between the receptor and the two donors substantially differed from the right to the left, which may be related to the asymmetry of the pyridine units in the DPP molecular structure.

### 2.3. UV-vis Absorption Spectra

**Figure 3:**
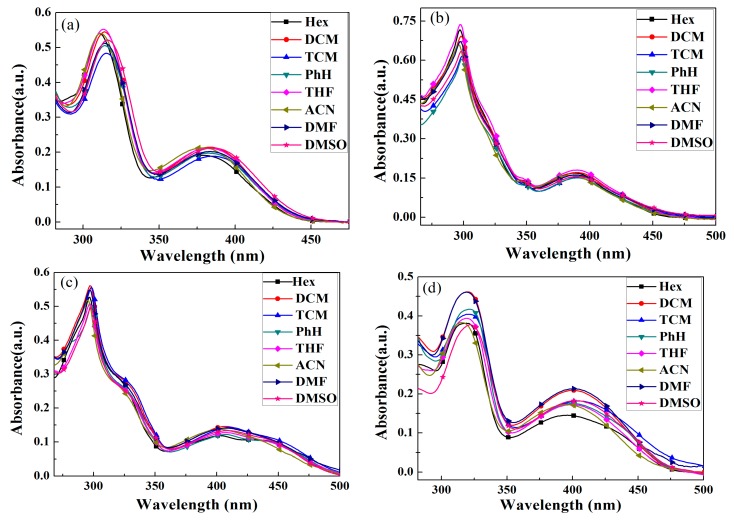
Ultraviolet-visible (UV-vis) absorption spectra of BPC-2DPx (**a**), BPC-3DPx (**b**), BPC-2DPP (**c**), and BPC-3DPP (**d**) in different solvents.

The UV-vis absorption spectra of the four compounds at a concentration of 1 × 10^−5^ mol/L solution of n-hexane, dichloromethane, chloroform, toluene, tetrahydrofuran, acetonitrile, N, N-dimethylformamide, and dimethyl sulfoxide are shown in Figure 3, and the corresponding absorption positions are shown in Table 1.

The UV-vis absorption spectra of the four compounds were similar because of their similar structures. In Figure 3, all four compounds had two significant characteristic absorption peaks in different solvents. The absorption peak between 300–340 nm is attributed to the π–π* electron transition of the conjugated skeleton, and the other peak (between 350–500 nm) is attributed to the ICT (π*–π*) between the electron donor BPC and the electron acceptor DPx or DPP. It can be seen that the absorption peak is not affected by solvent polarity (solvents are sorted according to their polarity [36] in ascending order). This insignificant solvatochromic effect is typical for the ground state of D–A compounds [12,37,38]. In the ground state, D–A-type compounds have lower polarity, and the effect of the solvent polarity on the electrons is insignificant [12,37,38], which results in a weak solvatochromic effect.

The substitution position strongly influences the locations of the compounds’ absorption peaks, as evident from the results that are summarized in Table 1. Comparing the absorption peaks of BPC-2DPx and BPC-3DPx, the two absorption bands shifted in opposite directions. The lower wavelength absorption band blue-shifted, and the higher wavelength absorption band red-shifted. Compared with the acceptor substituted at the two-position of the donor, its isomer, which the acceptor substituted at the two-position of the donor, processed better coplanarity between the donor and acceptor. Therefore, in the case of the three-position substitution, stronger interaction occurred between the donor and acceptor, which is more conducive to the occurrence of ICT and resulted in a red-shift of the longer wavelength absorption peak associated with π*–π* electron transfer. In addition, the decrease of electron density on the donor DPx ring caused by ICT resulted in a blue shift of the lower wavelength absorption peak.

When DPP was used as the acceptor, the change in the absorption band shift was also associated with the substitution position, but was opposite to that observed for DPx. Compared to DPx, the structure of a DPP is to replace a benzene ring on DPx with a pyridine ring. There is a sp_2_ hybridization orbital on the N atom of the pyridine ring, which is not involved in the bond forming, and is occupied by a pair of lone electrons, making the pyridine possess some basicity. So, the electronegativity of the N atom in the pyridine ring has a great influence on the density distribution of the electron cloud on the ring. The π electron cloud shifts to the N atom in the pyridine ring on the DPP unit; as a result, the density of the electron cloud around the N atom is higher than the carbon atoms in the pyridine ring, especially the carbon atoms on the ortho-position and para-position of the nitrogen atom. So, the pyridine molecule has less aromaticity than that of benzene. The above difference between DPx and DPP may have a different effect on the adjacent linked groups, and then explained the differences in the UV-vis spectra of DPP or DPx as the acceptor.

The effect of the acceptor on the absorption band of the compounds can be evaluated by comparing the absorption bands of BPC-2DPx and BPC-2DPP, as well as those of BPC-3DPx and BPC-3DPP in Table 1. For the BPC2-based compounds, when DPP was used as the acceptor instead of DPx, the resultant BPC-2DPP experienced a blue-shift for its high energy absorption and a red-shift for its low energy absorption, respectively. Compared with DPx, DPP had one more electron-deficient N atom, and thus had stronger electron-withdrawing ability, which is beneficial for ICT. Hence, the longer-wavelength absorption bands of the compounds with DPP as the acceptor exhibited red-shifts. In the case of the BPC3 as the donor unit, both absorption peaks of BPC-3DPP showed obvious red-shifts compared with those of BPC-3DPx. These shifts were due to the stronger electron-withdrawing ability of DPP.

The *E*_g_ values of the four compounds were calculated from their onset wavelength of the absorption (*E*_g_ = 1241/e_onset_ [13]), and the data are 2.82 eV, 2.70 eV, 2.48 eV, and 2.62 eV, respectively, for BPC-2DPx, BPC-3DPx, BPC-2DPP, and BPC-3DPP, which are dissolved in chloroform. The *E*_g_ values of the four compounds were much lower than the *E*_g_ of carbazole (3.2 eV) [39]. In the case of the compounds with DPP as the acceptor, which exhibited stronger electron-withdrawing ability than the acceptor of DPx, the *E*_g_ values of the compounds were smaller. Therefore, the introduction of a strong electron-withdrawing unit between two BPC units as the acceptor led to a lower band gap compared with carbazole derivatives. The substitution of an acceptor at the three-position of the donor is helpful to improve the coplanarity of the formed molecule, which is theoretically beneficial to reduce the *E*_g_ of the molecule.

### 2.4. Fluorescent Emission Spectra

**Figure 4:**
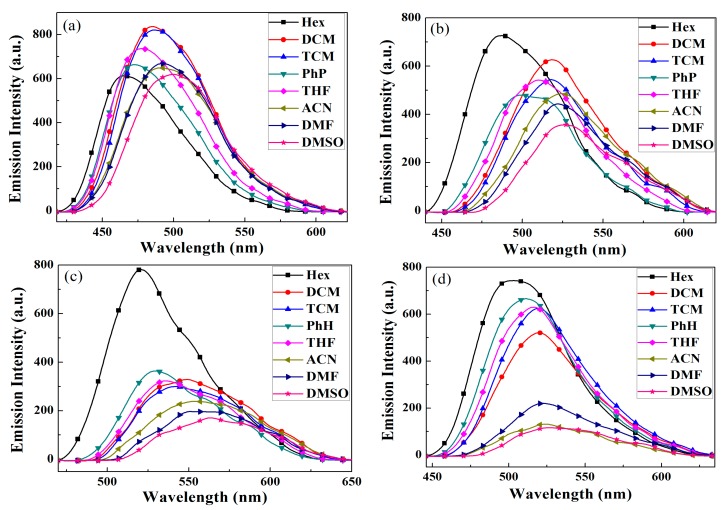
Fluorescent emission spectra of BPC-2DPx (**a**), BPC-3DPx (**b**), BPC-2DPP (**c**) and BPC-3DPP (**d**) in different solutions.

BPC-2DPx, BPC-3DPx, BPC-2DPP, and BPC-3DPP were excited at the maximum absorption peak to obtain the fluorescent emission spectra in eight different common solutions, as shown in Figure 4. The positions of the fluorescent emission peaks are shown in Table 2 (solvents are sorted according to their polarity [34] in ascending order).

Unlike the UV-vis absorption spectra, the fluorescent emission spectra of BPC-2DPx, BPC-3DPx, BPC-2DPP, and BPC-3DPP in each solution showed only one well-defined separate peak. In addition, their fluorescent emission peaks exhibited a very large Stokes shift (ranging from 86 nm to 153 nm) compared with the UV-vis absorption peaks. The results in Figure 4 show that the positions of the emission peaks of the four D–A–D compounds were substantially red-shifted with the increasing polarity of the solvent, except for anhydrous magnesium sulfate (ACN), but the trend remains unchanged. The fluorescent emission peaks of BPC-2DPx, BPC-3DPx, BPC-2DPP, and BPC-3DPP in DMSO were red-shifted 35 nm (from 465 nm to 500 nm), 42 nm (from 487 nm to 529 nm), 43 nm (from 520 nm to 563 nm), and 30 nm (from 503 nm to 533 nm), respectively, compared with those of the same compounds in n-hexane. These results demonstrate a strong solvatochromic effect. This phenomenon is easily explained. For a compound with a D–A structure, electrons in the excited state of the compound transfer from the HOMO to the LUMO, which increases the polarity of the excited state (S_1_). A more polar solvent is beneficial to the electron delocalization [12,37,38,40]. The excited state in the higher-polarity solvent can easily relax to a lower-energy state, which results in a red-shift of the fluorescent emission peak. The difference in the solvatochromic phenomena between the ground state and the excited states arises from the greater polarity of the excited states [12,37,38,40].

The fluorescent emission spectra in dichloromethane were used as an example. A comparison of the fluorescent emission spectra of BPC-2DPP and BPC-2DPx, which have different acceptors substituted at the same position on the donor, revealed that the fluorescent emission bands of the two compounds were 549 nm and 485 nm, respectively, and that the former was red-shifted by 64 nm compared with the latter. Similarly, a red-shift was also observed between the compounds of BPC-3DPP and BPC-3DPx. The red-shift trend of the fluorescent emission spectrum was the same as that observed in the UV-vis absorption spectra, and is attributed to the stronger electron-withdrawing effect of DPP than that of DPx, which strengthens the D–A interaction and facilitates the intramolecular charge transfer. The π*–π* electron transfer of the corresponding D–A–D structure compound was enhanced, resulting in a red-shift in the fluorescent emission spectrum.

The substitution position also affects the fluorescent emission spectrum of a compound. Upon comparing BPC-3DPx and BPC-2DPx, the fluorescent emission peaks were located at 518 nm and 485 nm (in dichloroform solution), respectively, where the former was red-shifted by 33 nm compared with the latter. When DPP was used as the acceptor, the peak in the fluorescent emission spectrum of BPC-3DPP was blue-shifted by 29 nm compared with that of BPC-2DPP, which is opposite to the substituent effect observed when DPx was used as the acceptor. The fluorescent emission spectra of these two groups of compounds were shifted in different directions, which is consistent with the variation rule of UV-vis spectra, and the reason is the same.

### 2.5. Cyclic Voltammetry

The electrochemical characteristics of four D–A–D compounds were studied by cyclic voltammetry (CV) using a 0.2 mol/L ACN solution of tetrabutylammonium hexafluoride as the electrolyte [13], and the HOMO and LUMO levels were estimated from the results.

**Figure 5:**
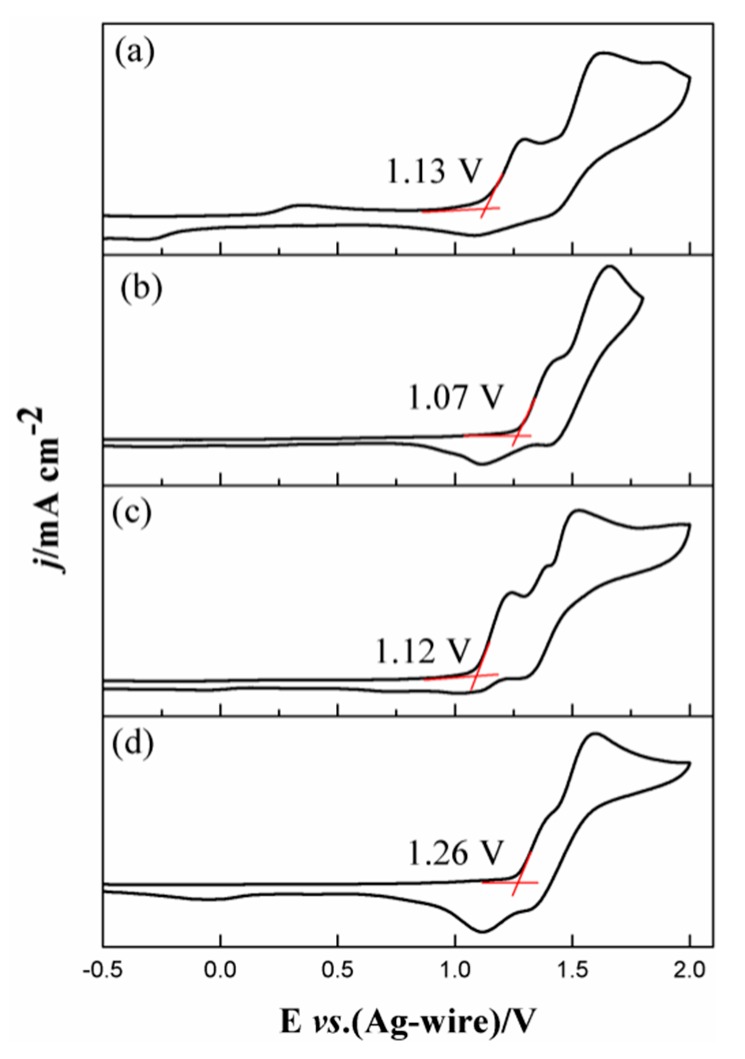
The cyclic voltammetry (CV) curves of BPC-2DPx (**a**), BPC-3DPx (**b**), BPC-2DPP (**c**), and BPC-3DPP (**d**).

The cyclic voltammograms (Figure 5) of the four compounds were similar, with two pairs of irreversible redox peaks. These peaks corresponded to the losing their electrons to form stable radical cations of the two electron donors (two benzocarbazoles) on each compound. The redox peaks of the compound BPC-2DPx were located at 1.59/1.33 V and 1.36/1.12 V, the redox peaks of BPC-3DPx were located at 1.52/1.32 V and 1.23/1.11 V, the redox peaks of BPC-2DPP were located at 1.62/1.40 V and 1.28/1.07 V, and the redox peaks of BPC-3DPP were located at 1.65/1.41 V and 1.39/1.11 V. The onset of the oxidation potentials (*E*_onset_) of BPC-2DPx, BPC-3DPx, BPC-2DPP, and BPC-3DPP were 1.13 V, 1.07 V, 1.12 V, and 1.26 V, respectively.

The HOMO levels (−5.55 eV, 5.49 eV, −5.54 eV, and −5.68 eV, respectively) of BPC-2DPx, BPC-3DPx, BPC-2DPP, and BPC-3DPP were calculated on the basis of the formula HOMO = −e (*E*_onset_ + 0.02 + 4.4) [13], where *E*_onset_ is the onset oxidation potential of the compounds. According to the equation LUMO = HOMO + *E*_g_, the LUMO energy levels of the compounds were calculated to be −2.73 eV, −2.79 eV, −3.06 eV, and −3.06 eV, respectively. Thus, the *E*_onset_ of the four compounds were similar, and they also had similar HOMO values. Compared with the LUMO levels of the two compounds with DPx as the acceptor, those of BPC-2DPP and BPC-3DPP were lower, which resulted in a higher ICT, a red-shift of the ICT absorption band, and a lower energy gap. The energy bands, LUMO/HOMO values, and other relevant data for the four compounds are summarized in Table 3.

The HOMO levels and the LUMO levels from the quantum calculations, as well as the *E*_g_ values deviated from the experimental values, are all presented in Table 3. The theoretical calculation values of *E*_g_ of the D–A–D compounds with DPP as the acceptor were smaller than those with DPx as the acceptor, which is consistent with the experimental values.

### 2.6. Thermostability Analysis

**Figure 6:**
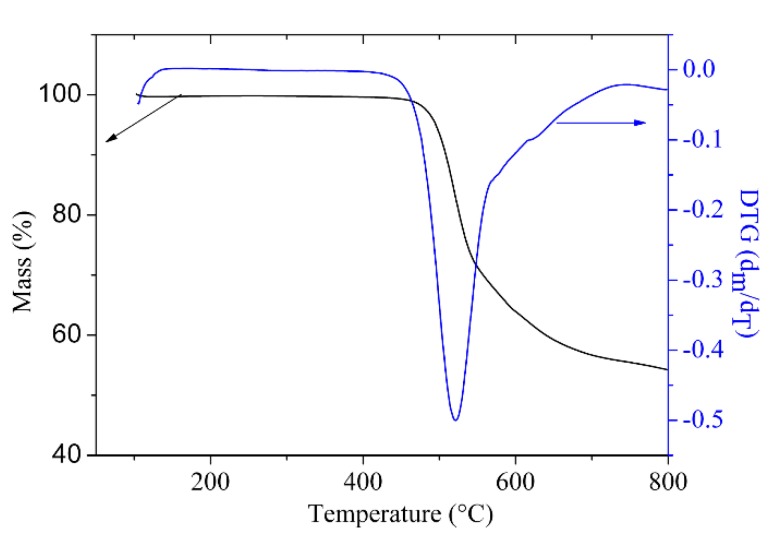
TGA curve of BPC-2DPx.

The thermal stability of BPC-2DPx was measured by thermogravimetric analysis (TGA) in an N_2_ atmosphere, and its TGA curves are shown in Figure 6. The TGA curves of BPC-3DPx, BPC-2DPP, and BPC-3DPP are shown in Figure S5a–c. These four compounds showed only a one-stage decomposition pattern. They all had a high thermal decomposition temperature (T_d_, defined as the temperature of the weightlessness was 5%), which were 445 °C, 448 °C, 453 °C, and 453 °C, respectively. Their maximum decomposition rates were 476 °C, 477 °C, 512 °C, and 510 °C, respectively, with very high thermal stability.

### 2.7. Performance of the Memory Devices

**Figure 7:**
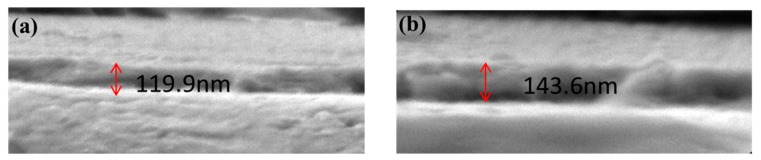
SEM images of a cross-sectional view of devices based on BPC-2DPx (**a**) and BPC-2DPP (**b**).

Figure 7 is the scanning electronic microscopic (SEM) image of the cross-sectional view of a memory device with the aim to determine the thickness of the organic layer. From bottom to top, the layers are the ITO (indium-tin oxide) glass, BPC-2DPx, or BPC-2DPP film, and the aluminum electrode, respectively. As can be seen from Figure 7, the thickness of the BPC-2DPx and BPC-2DPP films were 119.9 nm and 143.6 nm, respectively. The thin film is beneficial for the injection of holes and electrons during electrical storage [41,42]. The flatness of the top electrode also influences the performance of the device. The flatness of the top electrode can be controlled by the deposition rate and the time. The thickness can be controlled by the level of the vacuum and the deposition rate. The higher the vacuum, the more uniform the deposition rate. The aluminum top electrode was deposited under the pressure of 5.0 × 10^−4^ Pa; in this case, the deposition rate was maintained at about 5 nm/min. After 50 min, the thickness of the Al top electrode was about 230 nm.

**Figure 8:**
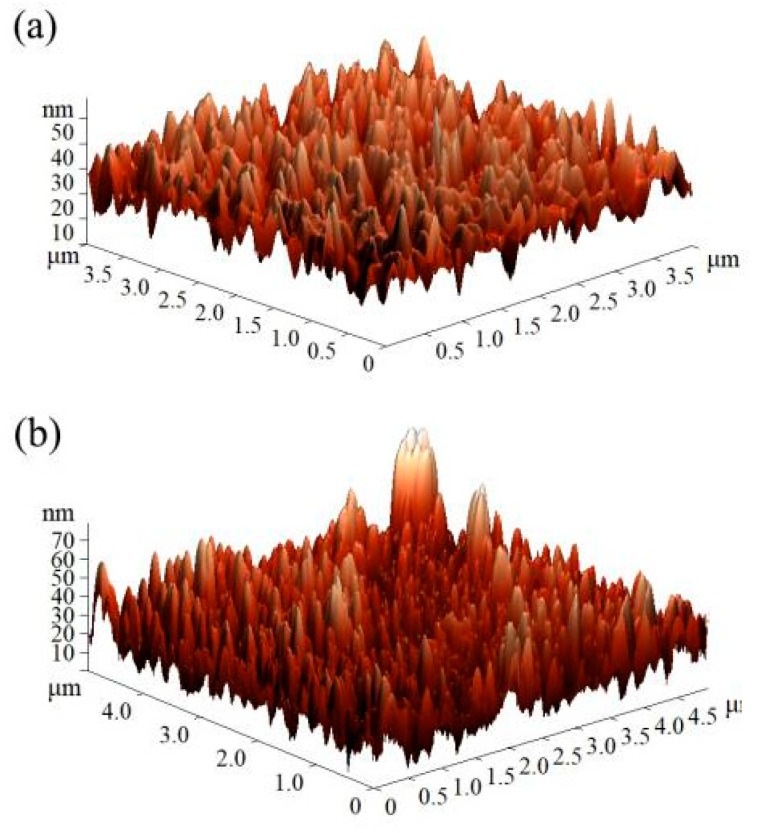
Three-dimensional (3D) atomic force microscopy (AFM) topography of BPC-2DPx (**a**) and BPC-2DPx (**b**) films.

The device performance is highly correlated with the surface morphology of the organic semiconducting active layer [42,43]. To investigate the surface root mean square roughness (RMS) of the films, atomic force microscopy (AFM) measurements were carried out on the BPC-2DPx and BPC-2DPP films. The 3D-AFM topography images are shown in Figure 8. The scanning range was 4.0 µm × 4.0 µm, and the distribution of the compound was relatively uniform. The RMS of BPC-2DPx film was 25.42 nm, while the RMS of BPC-2DPP film was 27.65 nm. The two films had high smoothness, which is favorable for the devices to exhibit good memory performance.

**Figure 9:**
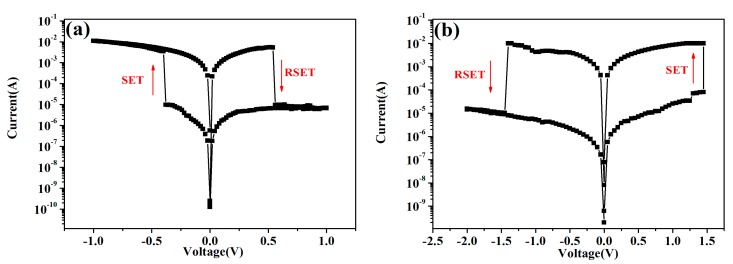
Current–voltage (I-V) characteristics of (**a**) ITO (Indium-Tin Oxide) glass/BPC-2DPx/Al and (**b**) ITO/BPC-2DPP/Al memory devices.

The memory behavior of the BPC-2DPx and BPC-2DPP films was demonstrated by the current−voltage (I−V) characteristics of ITO/BPC-2DPx/Al or ITO/BPC-2DPP/Al sandwich devices. Figure 9 shows the I–V electrical switching of the memory devices; the two devices present obvious bistability resistance characteristics. In the case of the device based on BPC-2DPx (Figure 9a), the voltage swept from −1 V to 1 V. In the negative scan from 0 V to −1 V, the device remained in the “OFF” or “0” state up to −0.38 V at first, at which the device was in a low-conductivity state. As the scan continued to the negative voltages, the current increased abruptly, and the device switched to the “ON” or “1” state, with the property of high conductivity. In the above process, the abrupt change in the current density represents the writing process of the device, and the ON/OFF switching ratio in current was about 10^3^. Then, during the continuous bias sweep, the device remained in the high conductivity state until the voltage increased to 0.54 V, at which point the current suddenly sharply decreased, and the device returned to the low-conductivity state, which can be regarded as the erasure process for data storage. Similarly, the scan voltage range of the device based on BPC-2DPP was −2 V–2 V, and the corresponding I−V curve is shown in Figure 9b. At the beginning, the device was in the OFF state. When the bias increased to about 1.4 V, that is, the threshold voltage, the current suddenly sharply increased and changed to the ON state, and the switching ratio was about 10^2^. The process of data writing was carried out. In the subsequent bias sweep, the device remained in the ON state. When the bias reached −1.4 V, the current decreased to the OFF state, and the switching ratio was about 10^3^.

The energy level differences at the interface of the bottom ITO electrode (−4.8 eV) and the deposited films of the compounds were calculated, and the data were 0.75 eV and 0.74 eV respectively, for ITO/BPC-2DPx and ITO/BPC-2DPP. At the same time, the energy level differences (energy barrier) at the interface of the LUMO level of the compounds and the top Al electrode (−4.28 eV) were also calculated; these were 1.55 eV (Al/BPC-2DPx) and 1.22 eV (Al/BPC-2DPP). The energy barrier between the interface of the ITO electrode and the organic layer indicated the hole injection barrier between the ITO electrode and these two layers. The electron injection barrier between the interface of the Al electrode and the organic thin films could also be obtained from the energy level difference between the Al electrode and these two layers. In this case, the hole injection barriers for both of the two devices are much lower than those of the electron injection barriers. Therefore, both organic layers have the p-type property, and the hole injection process dominates the writing and erasing process of the devices [41,42,43,44]. In D–A–D compounds, the electron acceptor cloud functions as carrier traps. When bias is applied between the electrodes, the carriers first fill the trap sites; after the traps were filled, an abrupt increase in current occurs, corresponding to the OFF to ON state transition. The existence of carrier traps results in the dipole formation, and an internal electric field is created, maintaining the high conductivity state. Under a reversed bias, the charge traps loses the charge, the internal electric field immediately disappears, and the device returns to its initial low conductivity state (OFF state), exhibiting binary non-volatile FLASH memory behavior [45]. From the view of the energy barriers discussed just as above, the ITO/BPC-2DPx/Al device should have a slightly higher switching voltage (the voltage from “ON” to “OFF” or the opposite change). However, the actual phenomenon is the opposite, i.e., the ITO/BPC-2DPx/Al device had a much lower switching voltage than that of the ITO/BPC-2DPP/Al, which suggested that the switching voltages are also determined by other factors besides the molecular structure of the organic layer. Compared with the other non-volatile memory device reported previously, these two devices still belong to the low-voltage driven devices, and have certain application prospects. The BPC3-based two other compounds are also used as the organic layer to construct the corresponding devices, which are designated to ITO/BPC-3DPx/Al and ITO/BPC-3DPP/Al, respectively. Their performance were also examined (Figure S6 in the supplementary information), and the data showed that the difference on substitution positions did not obviously affect the performance of the devices, which was different with its effect on the UV-vis absorption and fluorescence emission spectra. In view of this consideration, the in-depth analysis was not undertaken for getting the relationship between the performances of the four devices and the molecular structures of the four compounds.

**Figure 10:**
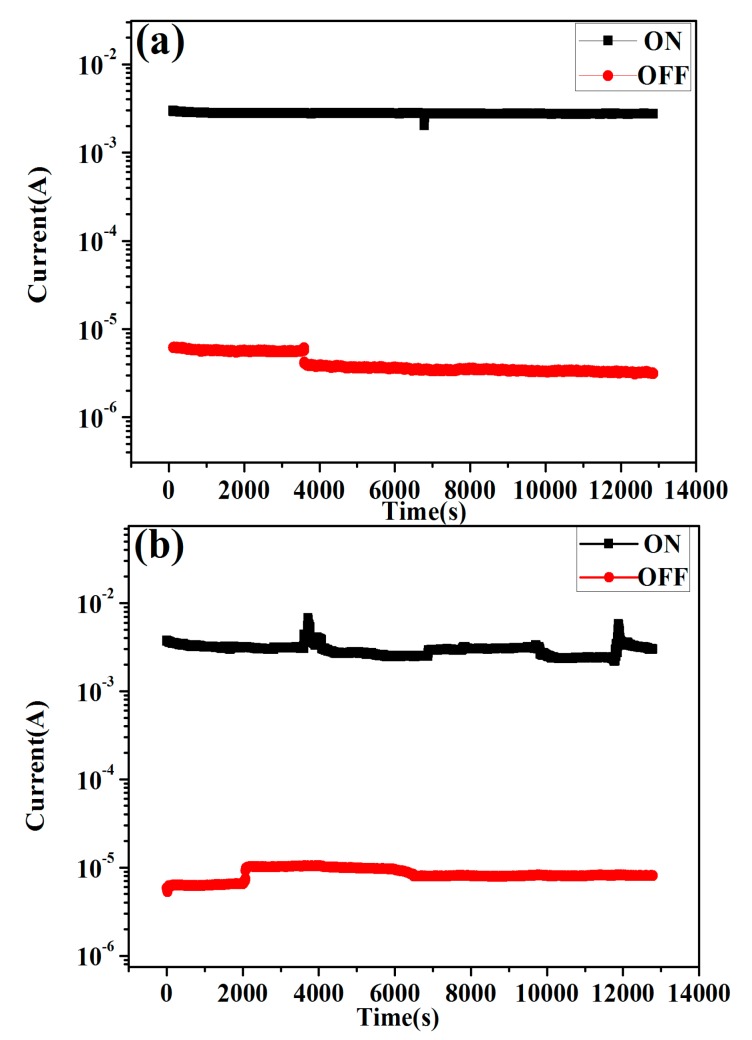
Retention time measurement for the ON and OFF states of the (**a**) ITO/BPC-2DPx/Al and (**b**) ITO/BPC-2DPP/Al devices under a under a continuous voltage of 0.5 V and 1 V, respectively.

The stability of data storage is an important index for evaluating the performance of memory devices. As shown in Figure 10, when a continuous read voltage of 0.5 V or 1 V was applied on the device of ITO/BPC-2DPx/Al or ITO/BPC-2DPP/Al, respectively, the current density of the device in ON and OFF states does not change significantly during the test time of 1300 s, revealing that the two devices possess good stability of data storage under ambient air conditions [41,43].

## 3. Experimental

### 3.1. Materials

4,4-dimethoxy benzil (98%), liquid bromine (99.0%), sodium borohydride (NaBH_4_, 98%), sodium carbonate (Na_2_CO_3_, 99.8%), 1-bromodecane (98%), anhydrous potassium carbonate (K_2_CO_3_, 99.8%), anhydrous magnesium sulfate, acetonitrile (ACN, AR), dichloromethane (DCM, AR), chloroform (TCM, AR), terahydrofuran (THF, AR), dimethyl formamide (DFM, AR), dimethyl sulfoxide (DMSO, AR), tetra-n-butyl bromide (99%), glacial acetic acid (AR), anhydrous ethanol (EtOH, 99.9%), toluene (PhH, AR), n-hexane (AR), tetra (triphenylphosphine) palladium (Pd(PPh_3_)_4_), and tetra-n-butylammonium fluoride (TBAF, AR) were purchased from Aladdin Industrial Corporation (Shanghai, China). 3,4-diaminopyridine, 4,7-dibromo-2,1,3-benzothiadiazole (98%), 9-phenyl-9*H*-carbazol-2-yl-boronic acid (98%, BPC2), or 9-phenyl-9*H*-carbazol-3-yl-boronic acid (98%, BPC3) were purchased from Aldrich Chemical Company and were used without further treatment. The thin layer chromatography silica gel (GF254) and the column chromatography silica gel (200-300 mesh) were provided by Qingdao Ocean Chemical Plant.

### 3.2. Characterization

^1^H and ^13^C NMR were measured by Varian AMX 400, where CDCl_3_ was used as the solvent and tetramethylsilane (TMS) was calibrated as the internal standard. MALDI-TOF MS was measured by Bruker Daltonics Flexanalysis. Elemental analysis was recorded by the Thermo Finnigan Flash EA 1112 analyzer. The thermal stability was measured by NETZSCH STA 499C TG-DSC in an N_2_ atmosphere, with a heating rate of 15 K/min. UV-vis data were collected by Varian Cary 5000. Fluorescent spectra were measured on an LS55 fluorescent photometer (Perkin Elmer). The electrochemical properties of the compounds were measured by workstation CHI 760C. A Canon Power Shot A3000 IS was used to take pictures of the four compounds and their solutions. SEM images were captured on a Hitachi S 4700 scanning electron microscope. Atomic force microscopy (AFM) measurements were performed using a MFP-3DTM instrument. Performance of the memory devices was tested with a Keithley 4200 instrument under ambient air conditions. Elemental analysis was performed on Thermo Finnigan Flash EA 1112 analyzer.

### 3.3. Synthesis

#### 3.3.1. Synthesis of the Acceptor Unit

The synthesis method of acceptor 2,3-bis(4-decyloxybenzene)-5,8-dibromoquinoxaline (compound **8**) can be found in the literature [35]. First, 4,7-dibromo-2,1,3-benzothiadiazole was suspended in anhydrous ethanol, and sodium borohydride was used as the reducing agent to get 3,6-dibromo-1,2-phenylenediamine (compound **2**) at a yield of 75% (Scheme 1a). Compound **7** was then prepared via the ether bond-breaking reaction in the presence of glacial acetic acid and 48% hydrogen bromide with 4,4-dimethoxybenzil (compound **5**) as the raw material, with a yield of 66% (Scheme 1c). Ultimately, compound **8** (yield 70%) was synthesized via a condensation reaction between compounds **2** and **7** (Scheme 1d). ^1^H NMR (CDCl_3_, 400 MHz, ppm): δ_H_ = 7.85 (s, 2H, ArH), 7.65 (dd, 4H), 6.88 (dd, 4H), 3.98 (t, 4H), 1.84–1.74 (m, 4H), 1.52–1.19 (m, 28H), 0.88 (t, 6H). ^13^C NMR (CDCl_3_, δ, ppm): δ = 160.39, 153.53, 138.94, 132.44, 131.63, 130.23, 123.36, 114.30, 68.04, 31.85, 29.53, 25.98, 22.65, 14.10.

The preparation of acceptor 2,3-bis(4-decyloxybenzene)-5,8-dibromopyrido[3,4-*b*]pyrazine (compound **9**) was described in the literature [39]. 3,4-diamino-2,5-dibromopyridine (compound **4**, yield 52%) was first synthesized by bromination of 3,4-diaminopyridine (Scheme 1b). Compound **9** (yield 63%) was then prepared via a condensation reaction between compounds **4** and **7** (Scheme 1e). ^1^H NMR (CDCl_3_, 400 MHz, ppm): δ_H_ = 8.68 (s, 1H, ArH), 7.66 (dd, 4H), 6.88 (dd, 4H), 3.99 (t, 4H), 1.86–1.73 (m, 4H), 1.48–1.20 (m, 28H), 0.88 (t, 6H). ^13^C NMR (CDCl_3_, δ, ppm): δ = 161.50, 156.77, 136.82, 132.14, 131.87, 129.80, 120.16, 114.75, 68.43, 32.11, 29.57, 26.24, 22.89, 14.32.

#### 3.3.2. Synthesis of the Four D–A–D Compounds

The four D–A–D compounds—BPC-2DPx, BPC-3DPx, BPC-2DPP, and BPC-3DPP—were obtained by the Suzuki coupling reaction (Scheme 2). Specifically, 3 g of BPC2, 3.93 g of compound 8, 40 mL of sodium carbonate solution (2 M), 60 mL of toluene, catalytic amount of Pd (PPh_3_)_4_ (5% of the molar mass of the reactants), and a little amount of the transfer agent TBAF were placed in a round-bottomed flask. The whole reaction system was heated at reflux under an argon atmosphere for 48 h. After completion of the reaction, the solvent was distilled under reduced pressure and washed twice with distilled water, and then extracted with chloroform. The organic phase was collected and dried over anhydrous magnesium sulfate to obtain the BPC-2DPx precursor. The precursor was separated and purified by silica-gel column chromatography using a mixed solution of *n*-hexane–dichloromethane (3:1 volume ratio) as the eluent to obtain a bright-yellow powdery solid of BPC-2DPx. Yield: 64%. ^1^H NMR (CDCl_3_, 400 MHz, ppm): δ = 8.29 (d, 2H, ArH), 8.23 (d, 2H), 8.08 (s, 2H), 7.95 (s, 2H), 7.75 (d, 2H), 7.65 (d, 4H), 7.54–7.38 (m, 14H), 7.34 (dd, 2H), 6.77 (d, 4H), 3.95 (t, 4H), 1.78 (m, 4H), 1.49–1.24 (m, 28H), 0.91 (t, 6H) (see Figure S1a in ESI). ^13^C NMR (CDCl_3_, δ, ppm): δ = 159.59, 151.00, 141.31, 140.60, 139.41, 138.54, 137.68, 136.19, 131.63, 131.33, 129.81, 129.75, 127.30, 127.18, 125.86, 123.28, 122.78, 122.61, 120.42, 119.90, 119.80, 114.02, 113.00, 109.72, 67.96, 31.88, 29.56, 29.54, 29.38, 29.31, 29.19, 26.01, 22.67, 14.13 (see Figure S1b in ESI). MS (MALDI-TOF): *m*/*z*: calcd for 1076.60; found 1076.66. Elemental analysis calcd (%): C 84.72, H 7.11, N 5.20, O 2.97; found: C 84.63, H 7.14, N 5.23, O 3.00.

The synthesis of BPC-3DPx was similar to that of BPC-2DPx. The precursor was separated and purified by silica-gel column chromatography using a mixed solution of n-hexane-dichloromethane (2:1 volume ratio) as eluent to obtain a yellow, powdery solid. Yield: 62%. ^1^H NMR (CDCl_3_, 400 MHz, ppm): δ= 8.77 (d, 2H, ArH), 8.28 (d, 2H), 7.99 (m, 4H), 7.67 (dd, 12H), 7.58 (d, 2H), 7.53–7.44 (m, 6H), 7.37 (m, 2H), 6.80 (d, 4H), 3.93 (t, 4H), 1.75 (m, 4H), 1.47–1.24 (m, 28H), 0.91 (t, 6H) (see Figure S2a in ESI). ^13^C NMR (CDCl_3_, δ, ppm): δ = 159.66, 150.42, 141.23, 140.42, 138.92, 138.42, 137.78, 131.65, 131.35, 130.38, 129.87, 129.43, 129.17, 127.39, 127.10, 125.81, 123.88, 123.28, 123.06, 120.24, 120.05, 114.12, 109.86, 109.17, 67.94, 31.88, 29.56, 29.54, 29.38, 29.31, 29.20, 26.00, 22.68, 14.13 (see Figure S2b in ESI). MS (MALDI-TOF): *m*/*z*: calcd for 1076.60; found 1076.63. Elemental analysis calcd (%): C 84.72, H 7.11, N 5.20, O 2.97; found: C 84.60, H 7.16, N 5.25, O 2.99.

The precursor of BPC-2DPP was similarly obtained. The precursor was separated and purified by silica-gel column chromatography. The eluent was a mixed solution of *n*-hexane–dichloromethane (2:1 volume ratio). After purification, an orange powdery solid was obtained. Yield: 65%. ^1^H NMR (CDCl_3_, 400 MHz, ppm): δ = 9.12 (s, 1H, ArH), 8.81 (d, ^1^H), 8.53 (dd, 1H), 8.31 (dd, 2H), 8.01 (dd, 1H), 7.74 (m, 4H), 7.69–7.45 (m, 17H), 7.39 (m, 2H), 6.86 (dd, 4H), 3.96 (q, 4H), 1.78 (m, 4H), 1.49–1.24 (m, 28H), 0.91 (m, 6H) (see Fig. S3a in ESI). ^13^C NMR (CDCl_3_,δ, ppm): δ = 160.41, 160.10, 157.79, 154.25, 151.75, 145.64, 141.52, 141.40, 141.28, 140.68, 137.65, 133.56, 131.57, 131.43, 131.33, 131.07, 130.78, 129.98, 129.89, 129.57, 128.87, 127.52, 127.50, 127.12, 126.00, 124.12, 123.72, 123.53, 123.29, 122.93, 120.42, 120.31, 114.33, 114.27, 109.94, 109.55, 109.22, 68.04, 31.88, 29.54, 29.37, 29.30, 29.19, 26.00, 22.67, 14.12 (see Figure S3b in ESI). MS (MALDI-TOF): m/z: calcd for 1077.59; found 1077.72. Elemental analysis calcd (%): C 83.53, H 7.01, N 6.49, O 2.97; found: C 83.40, H 7.07, N 6.55, O 2.98.

The precursor of BPC-3DPP was obtained by the same method used to prepare the precursor of BPC-2DPP. The precursor was separated and purified by silica-gel column chromatography. The eluent was a mixed solution of *n*-hexane–dichloromethane (1.5:1 volume ratio). After purification, a yellow, powdery solid was obtained. Yield: 70%. ^1^H NMR (CDCl_3_, 400 MHz, ppm): δ = 9.06 (s, 1H, ArH), 8.49 (s, 1H), 8.34 (t, 1H), 8.24 (m, 1H), 8.10 (d, 1H), 7.79 (t, 1H), 7.67 (t, 4H), 7.57–7.37 (m, 17H), 7.35 (dd, 2H), 6.79 (d, 4H), 3.97 (q, 4H), 1.80 (s, 4H), 1.50–1.24 (m, 28H), 0.88 (m, 6H) (see Figure S4a in ESI). ^13^C NMR (CDCl_3_, δ, ppm): δ = 160.32, 159.97, 158.57, 154.88, 152.42, 145.88, 141.62, 141.51, 141.37, 140.77, 140.50, 137.69, 137.59, 132.81, 131.89, 131.52, 131.25, 130.93, 130.77, 129.78, 127.35, 127.24, 127.15, 126.10, 124.00, 123.16, 122.43, 120.70, 120.51, 120.13, 119.82, 114.23, 114.16, 113.63, 112.94, 109.78, 68.04, 31.87, 29.54, 29.37, 29.29, 29.17, 26.01, 22.66, 14.11 (see Figure S4b in ESI). MS (MALDI-TOF): *m*/*z*: calcd for 1077.59; found 1077.72. Elemental analysis calcd (%): C 83.53, H 7.01, N 6.49, O 2.97; found: C 83.43, H 7.05, N 6.56, O 2.96.

### 3.4. Memory Device Fabrication

**Figure 11:**
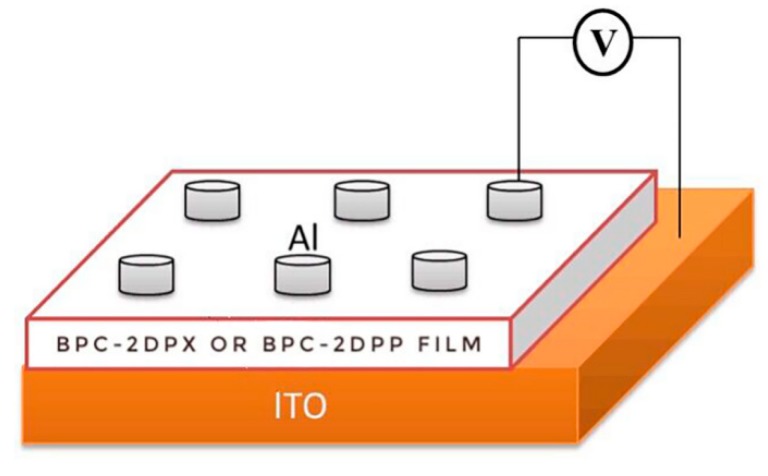
Scheme of the ITO/D-A-D compound/Al memory device.

A schematic sandwich structure comprised of a pair of electrodes and a layer of D–A–D compounds is shown in Figure 11. Firstly, the ITO glass was cleaned with water, and then sonicated in acetone and ethanol for 30 min, respectively. Then, the films of BDP-DPx and BDP-DPP were vapor-deposited on ITO glass under a pressure of around 1.0 × 10^−3^ Pa and at a temperature of 185 °C. Finally, the Al top electrode was thermally evaporated onto the film surface through a shadow mask with a thickness around 100 nm and area of 0.20 mm^2^ at a pressure of 5.0 × 10^−4^ Pa with a uniform depositing rate of 4 nm/min.

## 4. Conclusions

Four D–A–D compounds were synthesized using a one-step Suzuki coupling reaction with DPx or DPP as the acceptor, and BPC2 or BPC3 as the donor. The acceptor DPP had one more electron-deficient N-atom than DPx, which resulted in a larger red-shift of both the ICT absorption band and the fluorescent emission spectrum of compounds with DPP as the acceptor compared with those of the compounds with DPx as the acceptor, indicating that the ICT effect between DPP and the donor is stronger than that between DPx and the donor. The molecules obtained with BPC3 as the donor exhibited greater coplanarity than those with BPC2 as the donor. When DPx was used as the acceptor, more significant red-shifts occurred for both the ICT absorption band and the fluorescent emission spectra of the BPC3-based compounds compared with those of the BPC2-based compounds. When DPP was used as the acceptor, a larger blue-shift occurred for both the ICT absorption band and the fluorescent emission spectra of the BPC3-based compounds compared with those of the BPC2-based compounds. The replacement of the benzene ring with the pyridine ring in the DPP unit alters the direction of the electron cloud density of the DPx unit, and then leads to the different substituent effect with the adjacent linked groups. The *E*_g_ values of the four D–A–D compounds—BPC-2DPx, BPC-3DPx, BPC-2DPP, and BPC-3DPP—were 2.82 eV, 2.70 eV, 2.48 eV, and 2.62 eV, respectively. In summary, compared with DPx, DPP exhibits stronger electron-withdrawing ability. When used for constructing D–A–D compounds with the same donor, molecules with DPP as the electron acceptor have lower *E*_g_ values. Better coplanarity between the donor and acceptor is beneficial for improving the degree of conjugation, and in theory may reduce the *E*_g_ value. This discovery aids in adjusting the *E*_g_ of optoelectronic materials by enabling the selection of an appropriate donor and acceptor moiety during the construction of the D–A–D compound to meet different application requirements. The memory devices fabricated with BPC-2DPx and BPC-2DPP as the active material were found to exhibit a binary non-volatile flash memory nature. They also exhibited a high ON/OFF current ratio and respectable retention ability under ambient conditions.

## Figures and Tables

**Table 1:** The max absorption wavelengths (λ_max_, nm) of BPC-2DPx, BPC-3DPx, BPC-2DPP, and BPC-3DPP in different solvents.

Solvent	BPC-2DPx	BPC-3DPx	BPC-2DPP	BPC-3DPP
Hex	311, 379	297, 388	296, 403	319, 396
PhH	314, 383	299, 390	298, 408	321, 399
THF	313, 383	298, 390	297, 408	319, 399
DCM	314, 383	298, 390	297, 408	320, 400
TCM	315, 384	299, 391	299, 410	320, 403
DMF	314, 383	297, 389	297, 407	319, 401
DMSO	316, 385	298, 391	298, 410	322, 403
ACN	312, 378	296, 386	295, 402	316, 395

**Table 2:** The maximum fluorescent emission wavelengths (λ_max_, nm) of BPC-2DPx, BPC-3DPx, BPC-2DPP, and BPC-3DPP in different solvents.

Solvent	BPC-2DPx	BPC-3DPx	BPC-2DPP	BPC-3DPP
Hex	465	487	520	503
PhH	473	500	530	511
THF	478	510	536	517
DCM	485	518	549	520
TCM	486	518	542	519
DMF	492	522	551	522
DMSO	500	529	563	533
ACN	490	525	553	524

**Table 3:** The electrochemical and optical property data of compounds, include dihedral angles, *E*_onset_, λ_max_, λ_onset_, *E*_g_, highest occupied molecular orbital (HOMO), and lowest unoccupied molecular orbital (LUMO).

	Dihedral Angles (deg)	λ_max_ (nm)/λ_onset_ (nm)	*E*_g_^b^ (eV)	HOMO ^c^ (eV)	LUMO ^d^ (eV)	*E*_g_^e^ (eV)	HOMO ^e^ (eV)	LUMO ^e^ (eV)
BPC-2DPx	41.77, 40.92	314, 383/440	2.82	−5.55	−2.73	3.32	−5.37	−2.05
BPC-3DPx	37.28, 36.86	298, 390/459	2.70	−5.49	−2.79	3.09	−5.09	−2.00
BPC-2DPP	39.42, 19.42	297, 408/501	2.48	−5.54	−3.06	2.94	−5.35	−2.41
BPC-3DPP	36.21, 15.23	320, 400/473	2.62	−5.68	−3.06	2.80	−5.13	−2.33

^a^ Dihedral angle between the central acceptor and the adjacent benzene ring. ^b^ Band gap calculated from *E*_onset_, *E*_g_ = 1241/*E*_onset_. ^c^ Calculated from *E*_onset_ of the compounds, HOMO = −e(*E*_onset_ + 0.02 + 4.4). ^d^ Estimated using empirical equations LUMO = HOMO + *E*_g_. ^e^ *E*_g_, HOMO and LUMO were calculated based on the density functional theory (DFT).

## Supplementary Materials

The following are available online at http://www.mdpi.com/1996-1944/11/10/2063/s1, Figure S1. (a) ^1^H NMR spectrum of BPC-2DPx in CDCl_3_ solution (b) ^13^C NMR spectrum of BPC-2DPx in CDCl_3_ solution, Figure S2. (a) ^1^H NMR spectrum of BPC-3DPx in CDCl_3_ solution, (b) ^13^C NMR spectrum of BPC-3DPx in CDCl_3_ solution, Figure S3. (a) ^1^H NMR spectrum of BPC-2DPP in CDCl_3_ solution, (b) ^13^C NMR spectrum of BPC-2DPP in CDCl_3_ solution, Figure S4. (a) ^1^H NMR spectrum of BPC-3DPP in CDCl_3_ solution, (b) ^13^C NMR spectrum of BPC-3DPP in CDCl_3_ solution, Figure S5. (a) TGA curves of BPC-3DPx, (b) TGA curves of BPC-2DPP, (c) TGA curves of BPC-3DPP, Figure S6. Current-voltage (I-V) characteristics of (a) ITO/BPC-3DPx/Al and (b) ITO/BPC-3DPP/Al memory devices.
