# Supplementary material for: Effects of Pyrazine Derivatives and Substituted Positions on the Photoelectric Properties and Electromemory Performance of D–A–D Series Compounds
Source: Materials (Basel). 2018 Oct 22;11(10):2063. doi: 10.3390/ma11102063 (PMC6213557; doi:10.3390/ma11102063)
Supplement: Supplementary file 1 [file materials-11-02063-s001.pdf]

Electronic Supplementary Information

Effects of Pyrazine Derivatives and Substituted Positions on the Photoelectric Properties and Electro-memory Performance of D-A-D Series compounds

Xuejing Song, Lingqian Kong, Hongmei Du, Xiangyu Li, Hanlin Feng, Jinsheng Zhao and Yu Xie

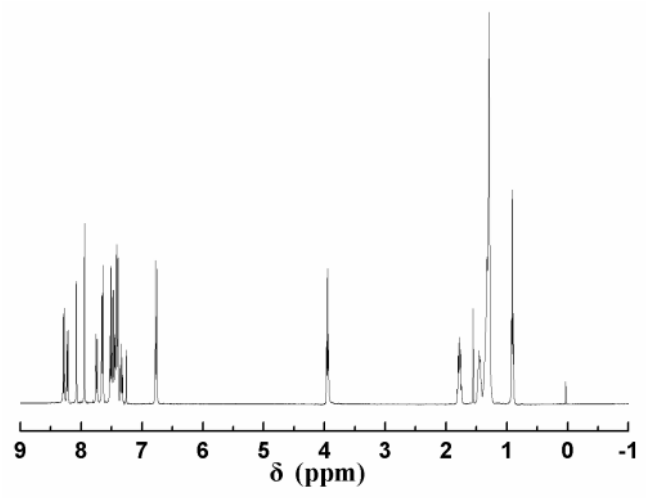

(a)

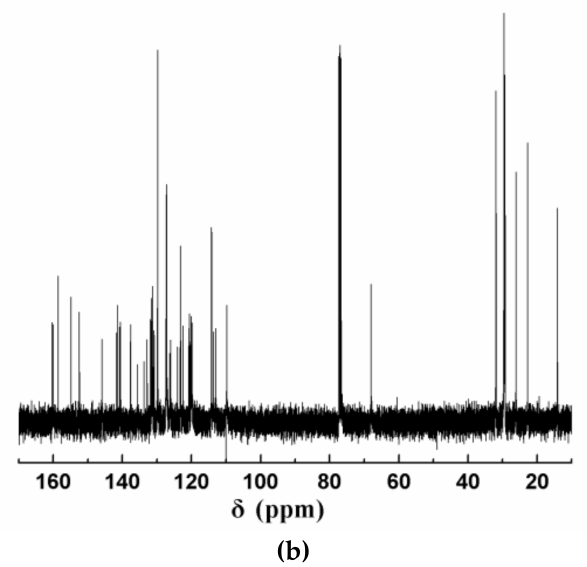

(b)

Figure S1. (**a**) ^{1}H NMR spectrum of BPC-2DPx in $CDCl_3$ solution (**b**) ^{13}C NMR spectrum of BPC-2DPx in $CDCl_3$ solution.

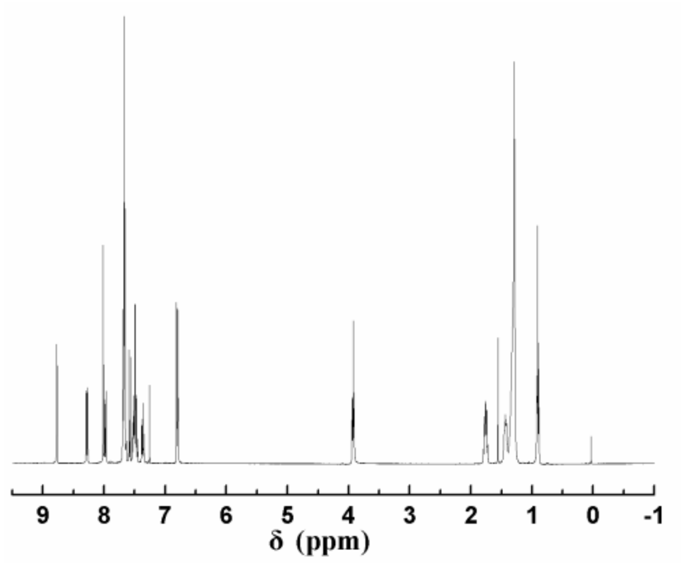

(a)

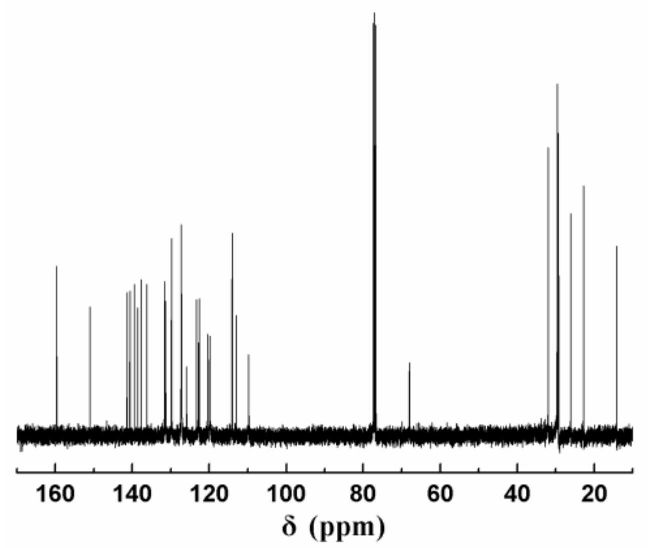

(b)

Figure S2. (**a**) ^{1}H NMR spectrum of BPC-3DPx in $CDCl_3$ solution, (**b**) ^{13}C NMR spectrum of BPC-3DPx in $CDCl_3$ solution**.**

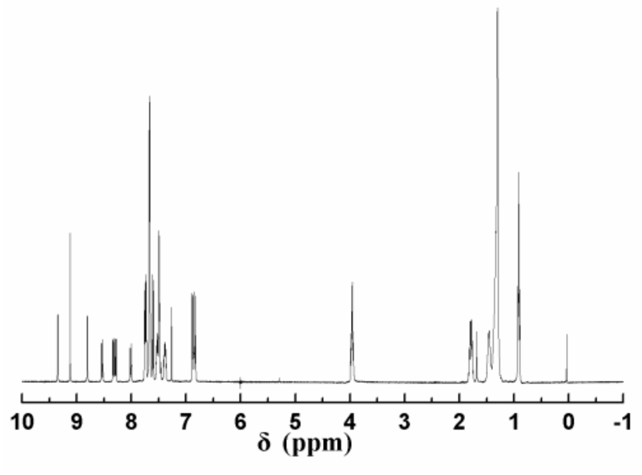

(**a**)

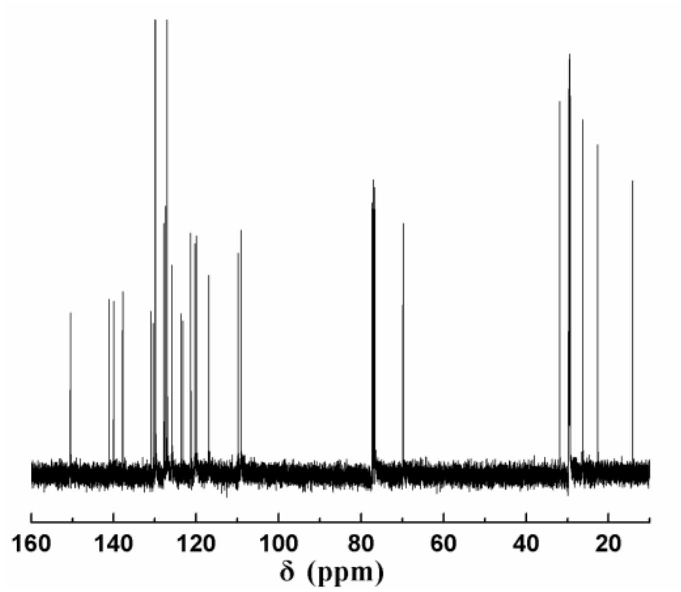

(**b**)

Figure S3. (**a**) ¹H NMR spectrum of BPC-2DPP in $CDCl_3$ solution, (**b**) ¹³C NMR spectrum of BPC-2DPP in $CDCl_3$ solution.

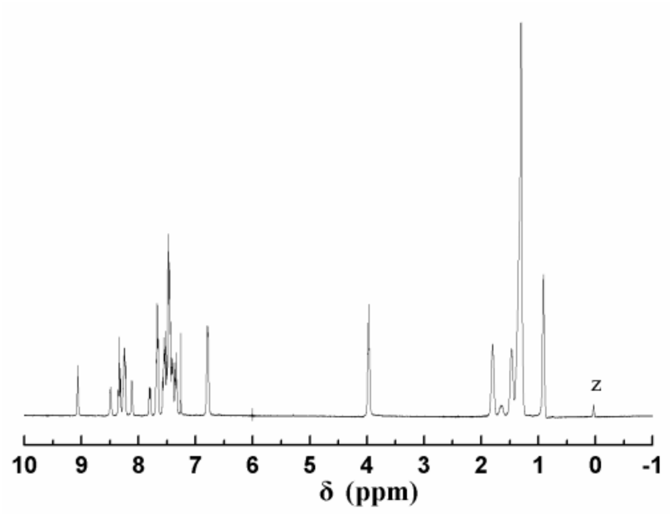

(**a**)

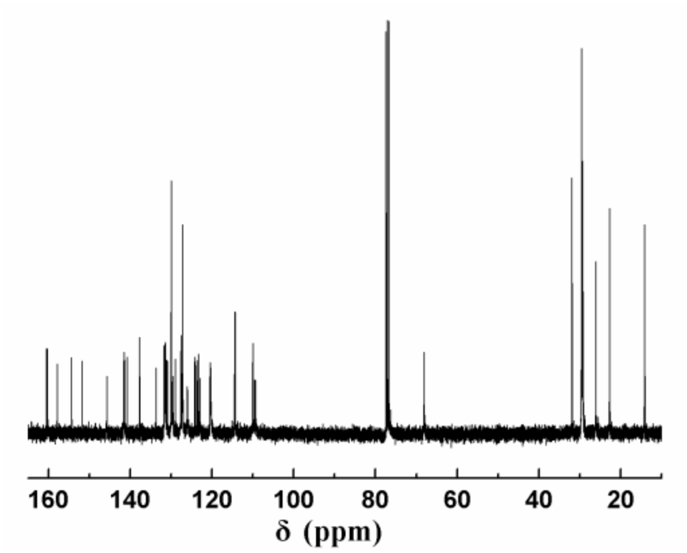

(**b**)

Figure S4. (**a**) ^{1}H NMR spectrum of BPC-3DPP in $CDCl_3$ solution, (**b**) ^{13}C NMR spectrum of BPC-3DPP in $CDCl_3$ solution**.**

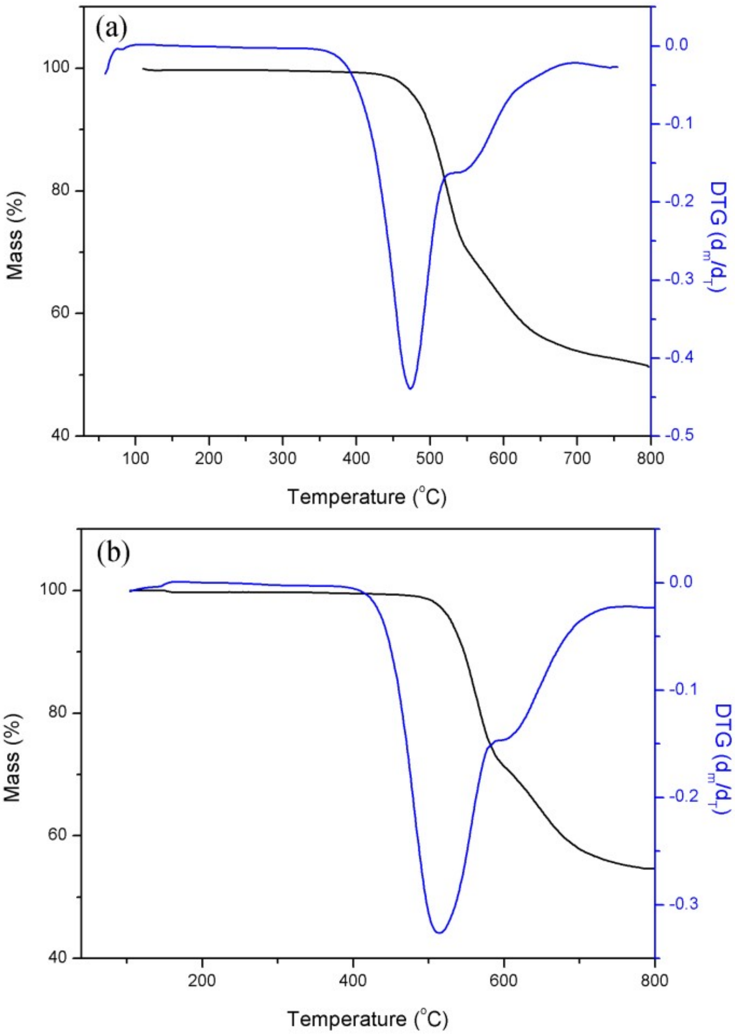

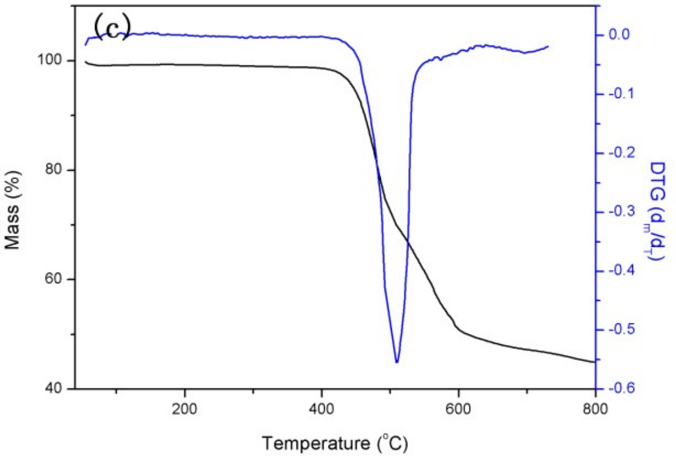

Figure S5. (**a**) TGA curves of BPC-3DPx, (**b**) TGA curves of BPC-2DPP, (**c**) TGA curves of BPC-3DPP.

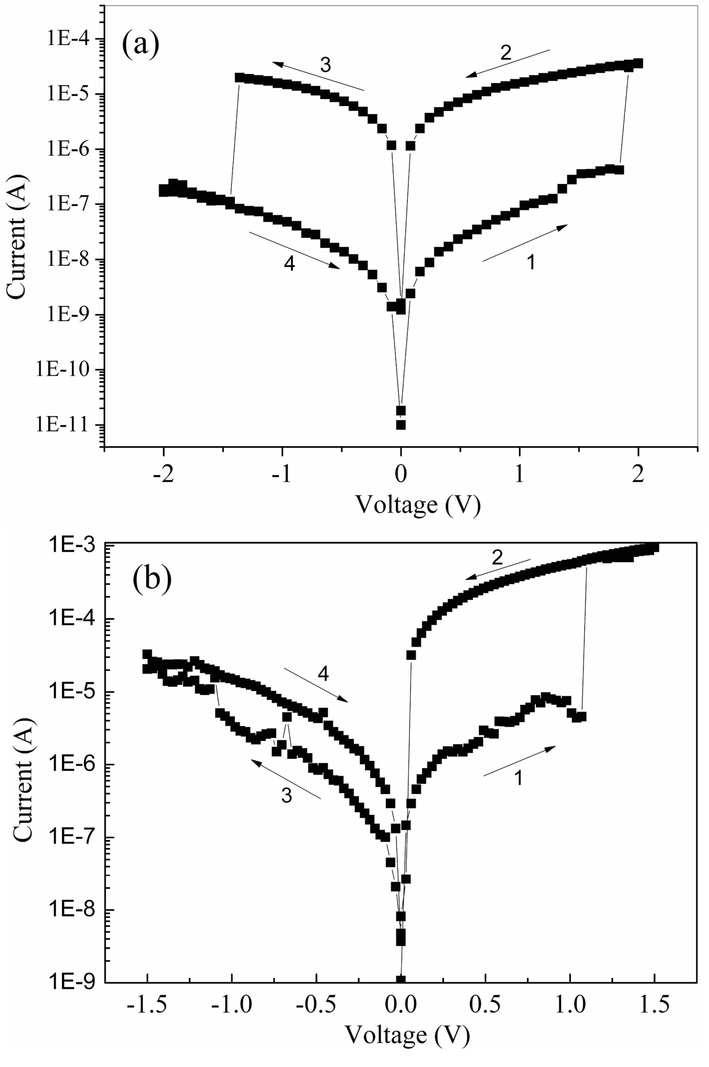

Figure S6. Current-voltage (I-V) characteristics of (**a**) ITO/BPC-3DPx/Al and (**b**) ITO/BPC-3DPP/Al memory devices.
